# Supplementary figures and images for: Morphogenesis in Trypanosoma cruzi epimastigotes proceeds via a highly asymmetric cell division
Source: PLoS Negl Trop Dis. 2023 Nov 2;17(11):e0011731. doi: 10.1371/journal.pntd.0011731 (PMC10656021; doi:10.1371/journal.pntd.0011731)

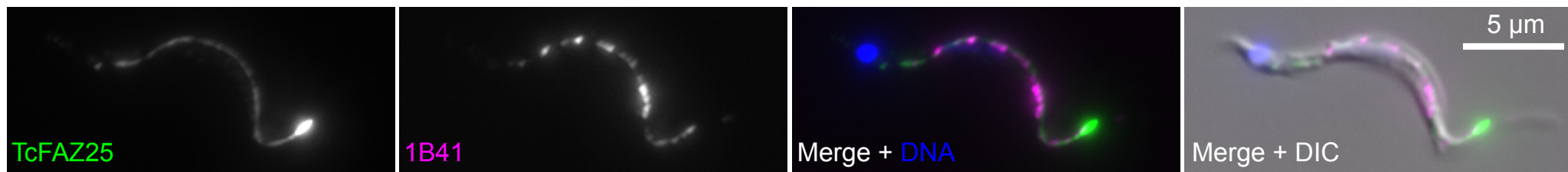

S1 Fig

Supplement: S1 Fig — T. cruzi cells expressing 3xTy1-mNeonGreen::TcFAZ25 were fixed, then labeled with 1B41 (1B41, magenta) Ty1 to label TcFAZ25 (TcFAZ25, green) and then imaged using epifluorescence microscopy. Several trypomastigote form parasites were present in the epimastigote cultures. (PDF) [file pntd.0011731.s001.pdf]

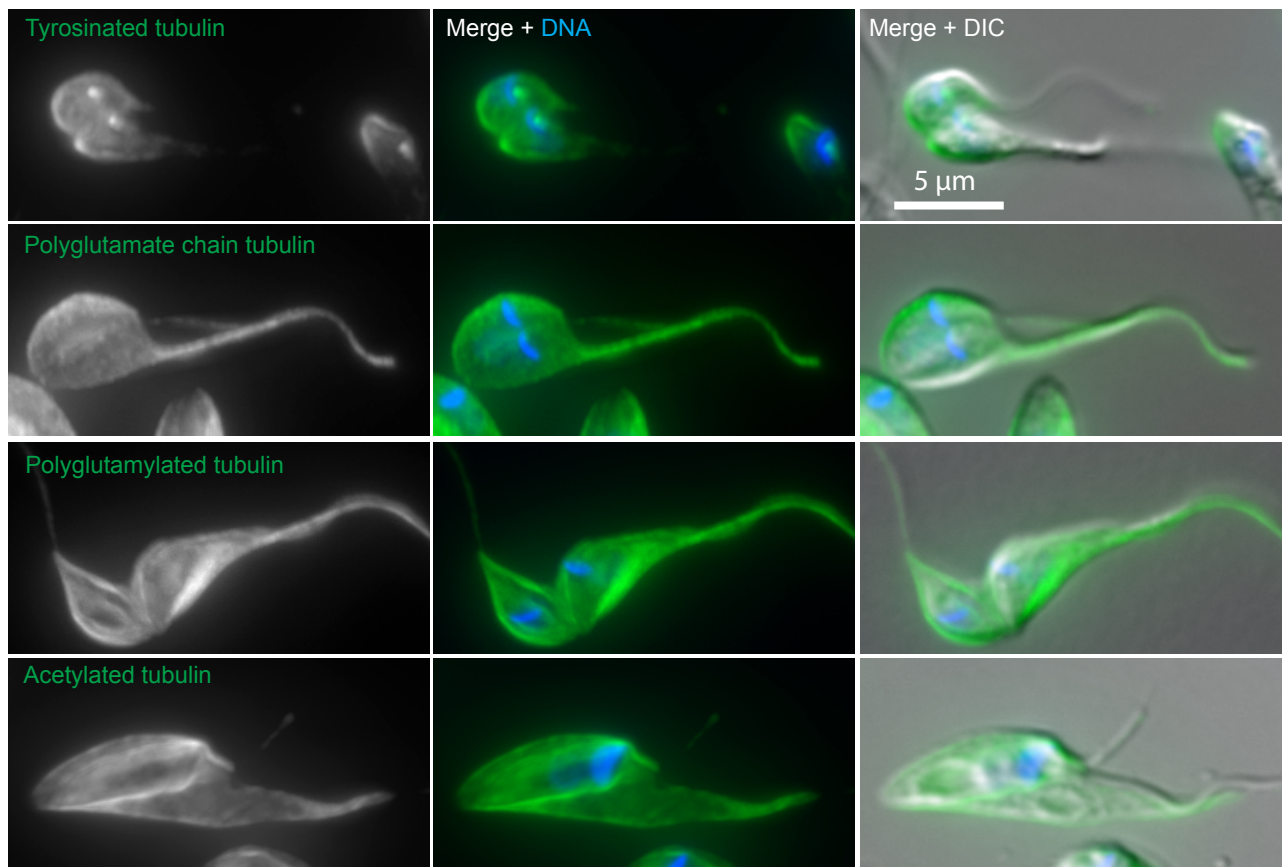

S2 Fig

Supplement: S2 Fig — T. cruzi cells were fixed, then labeled with anti-tyrosinated tubulin (YL1/2), polyglutamylated chain tubulin (GT335), polyglutamylated tubulin (IN105), or acetylated tubulin (6-11B-1), followed by secondary antibodies conjugated to Alexa488, and DAPI to label DNA. Cells were then imaged using epifluorescence and DIC microscopy. (PDF) [file pntd.0011731.s002.pdf]

A

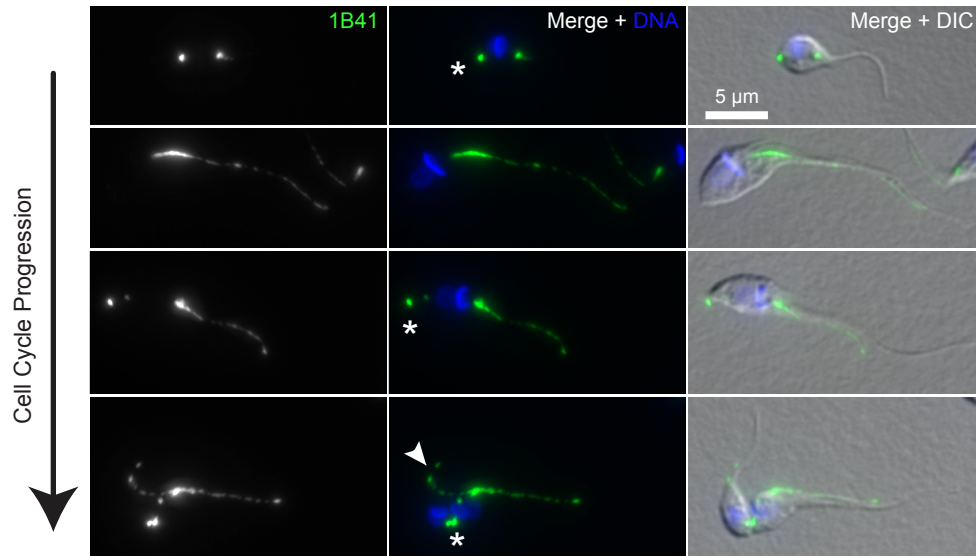

B

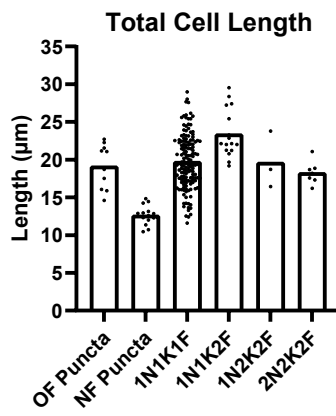

C

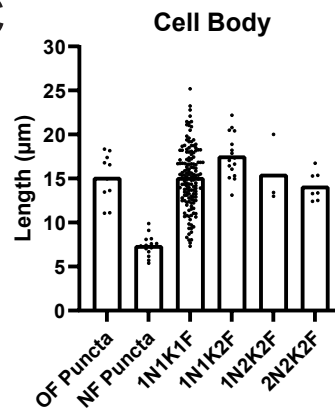

D

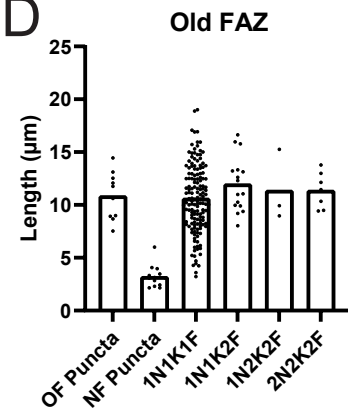

E

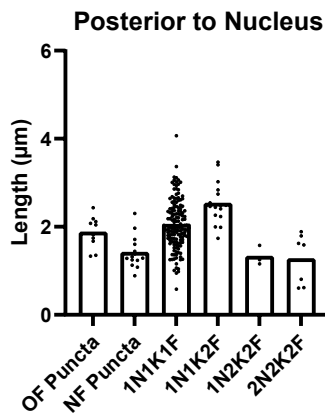

F

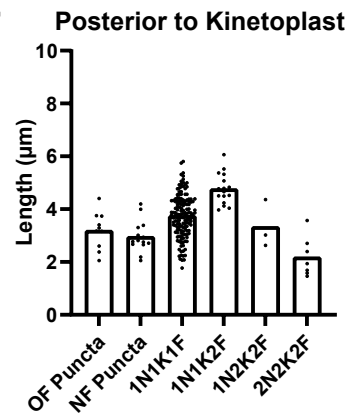

G

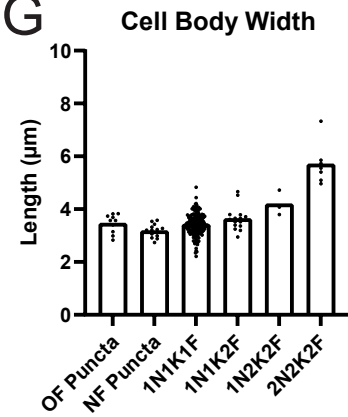

H

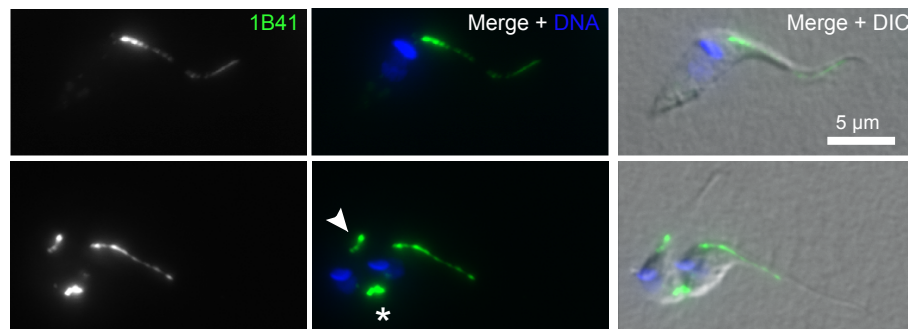

Supplement: S3 Fig — (A) The parental T. cruzi Y strain used for making all the tagged cell lines in this work was fixed and then labeled with 1B41 antibody, followed by fluorescently conjugated secondary antibodies and DAPI to label the DNA. The cells were imaged using immunofluorescence and DIC microscopy. Asterisks highlight the 1B41 posterior punctum labeling, while the arrowheads identify the new FAZ. (B) Measurements of the total cell length as defined in Fig 4A. (C) Measurements of the cell body length as defined in Fig 4A (D) Measurements of the Old FAZ length as defined in Fig 4A (E) Measurements of the posterior to nucleus distance as defined in Fig 4A. (F) Measurements of the posterior to kinetoplast distance as defined in Fig 4A (G) Measurements of the cell body with as defined in Fig 4A. (H) T. cruzi Brazil A4 strain cells were fixed and labeled with 1B41, followed by fluorescently conjugated secondary antibodies and DAPI to label the DNA. The cells were then imaged using immunofluorescence microscopy. (PDF) [file pntd.0011731.s003.pdf]

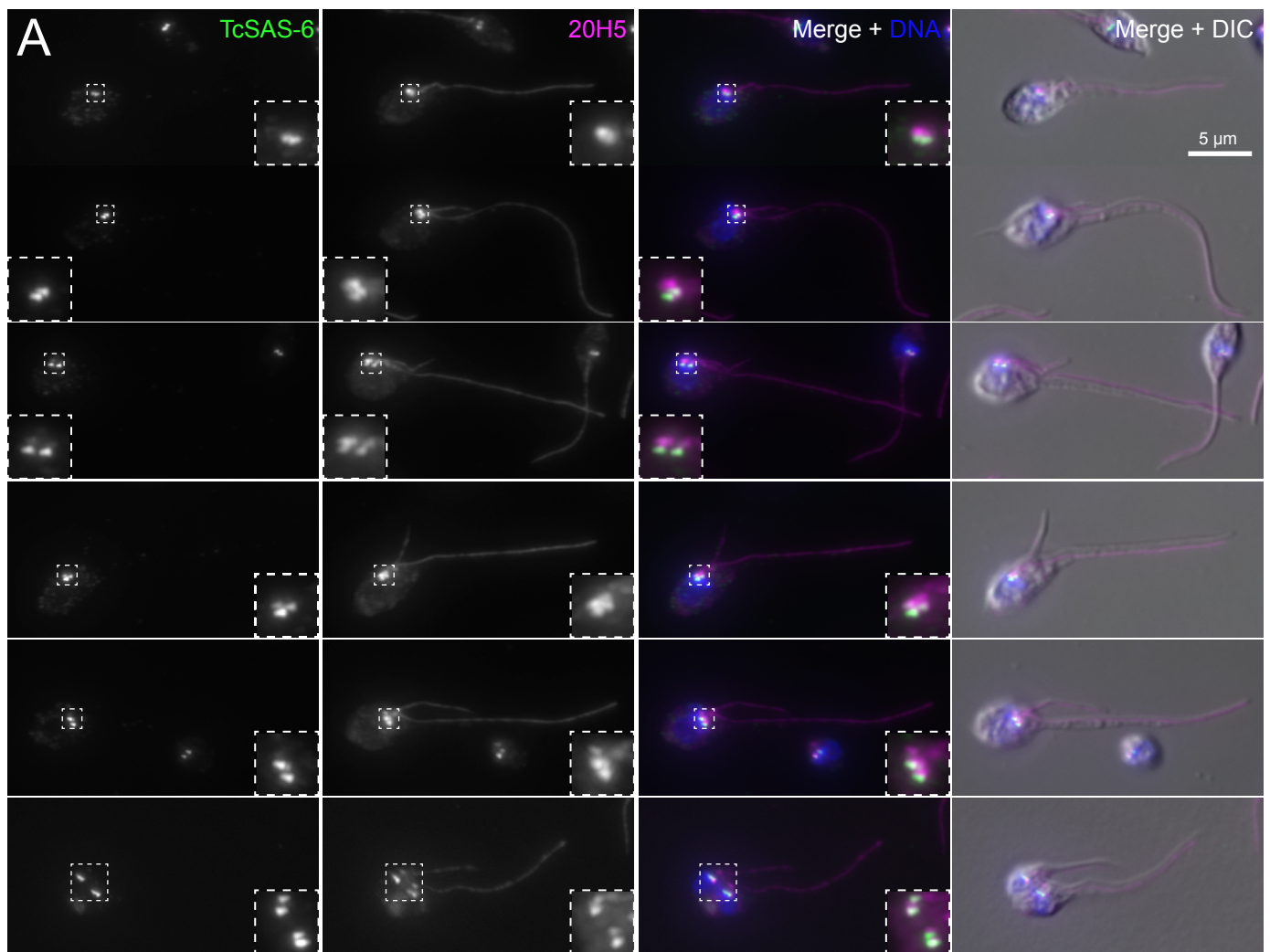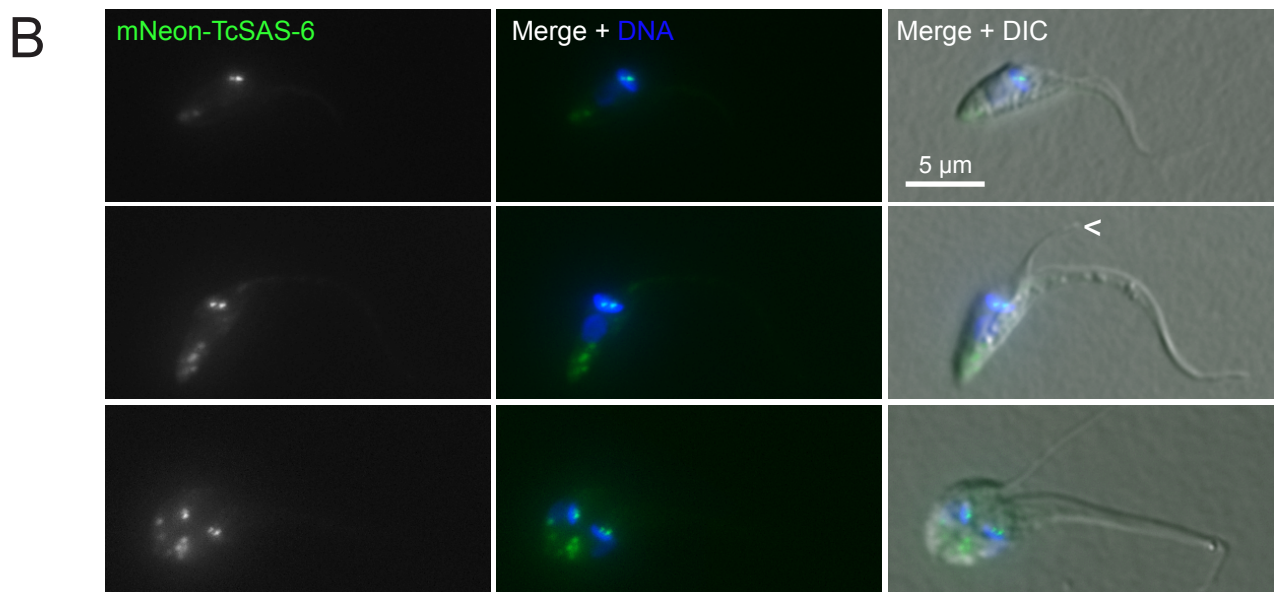

S4 Fig

Supplement: S4 Fig — (A) Cells carrying a 3xTy1-mNeonGreen::TcSAS-6 allele were fixed and labeled with anti-Ty1 and 20H5 to label SAS-6 and TcCentrin2, followed by fluorescently labelled secondary antibodies and DAPI to label DNA. The cells were then imaged using immunofluorescence microscopy. Insets show a 3X magnification of the basal body regions. (B) Cells carrying a 3xTy1-mNeonGreen::TcSAS-6 allele were fixed and then incubated with DAPI to label the DNA. The innate mNeonGreen fluorescence was then imaged using epifluorescence microscopy. (PDF) [file pntd.0011731.s004.pdf]

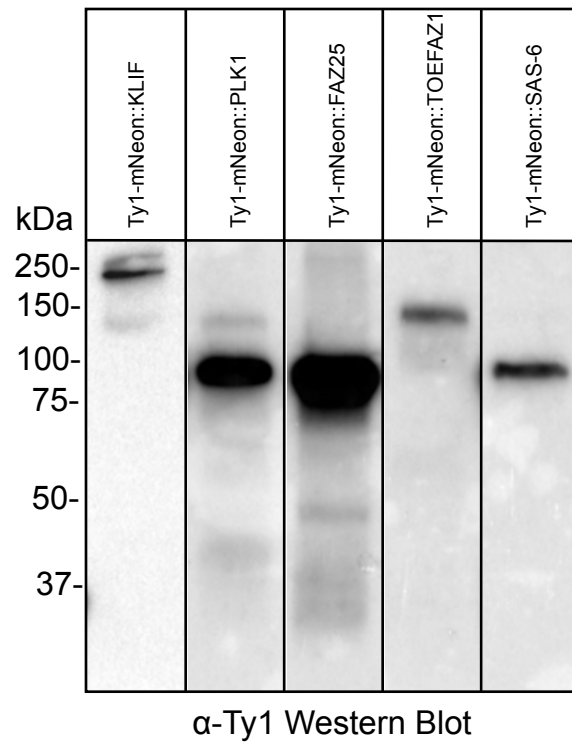

S5 Fig

Supplement: S5 Fig — The KLIF, PLK, FAZ25, TOEFAZ1, and SAS-6 cell lines endogenously tagged with 3X Ty1-mNeonGreen were harvested and then lysed in SDS-PAGE loading buffer, followed by fractionation using SDS-PAGE. Fractionated lysates were transferred to nitrocellulose and probed with anti-Ty1 antibody. (PDF) [file pntd.0011731.s005.pdf]
